# Retraction of: Apigenin inhibits growth and migration of fibroblasts by suppressing FAK signaling

**DOI:** 10.18632/aging.206230

**Published:** 2025-03-31

**Authors:** Hongyi Wang, Bingyu Guo, Shixiu Lin, Peng Chang, Kai Tao

**Affiliations:** 1 Reconstructive and Plastic Surgery, General Hospital of North Theater, PLA, Shenyang, P.R. China

**Keywords:** apigenin, hypertrophic scar, FAK, phosphorylation

**This article has been retracted:** Aging has completed its investigation of this paper. We found multiple instances of image reuse and overlap with images from unrelated papers from different institutions. Specifically:

**Figure 1:**

The transwell assay image in Figure 1G is a reuse of image in Figure 4G from the authors earlier paper [1];

The control transwell assay image in Figure 1H was reused in Figure 2G of the authors 2021 paper [2], which is already retracted;

The control transwell assay image in Figure 1H overlaps with an unrelated image in Figure 5D from already retracted paper [3];

The images in Figure 1H, 1I overlap with the images in Figure 5B from earlier published unrelated paper [4];

Also, images in Figure 1I overlap with the images in Figures 4A and 5B from already retracted paper [5];

Additionally, the images in Figure 1I were found in Figure 3F from concurrently published paper [6].

**Figure 4:**

The transwell assay images in Figure 4A, 4B were found to overlap with images in Figure 4F of [1], Figure 4C of [4], Figure 4D of [5], and Figure 3C from [6].

The corresponding author informed us that these overlaps were due to improper data storage in the laboratory and has applied for a retraction. Given these findings, the Editorial decision was to retract the paper. All authors have agreed to this retraction.
